# Serum miR-101-3p combined with pepsinogen contributes to the early diagnosis of gastric cancer

**DOI:** 10.1186/s12881-020-0967-8

**Published:** 2020-02-10

**Authors:** Weiwei Zeng, Shuxiang Zhang, Lei Yang, Wenchao Wei, Jie Gao, Ni Guo, Fengting Wu

**Affiliations:** Department of Gastroenterology, Dongying People’s Hospital, No. 317, Chengnan First Road, Dongying City, 257091 Shandong Province China

**Keywords:** Gastric cancer, Atrophic gastritis, MiR-101-3p, Pepsinogen, Diagnostic marker

## Abstract

**Background:**

This study aimed to explore the diagnostic value of serum miR-101-3p combined with pepsinogen (PG) on early diagnosis of gastric cancer (GC).

**Methods:**

A total of 61 atrophic gastritis (AG) and 86 GC patients, and 50 healthy volunteers were enrolled. The serum expression of miR-101-3p was measured by qRT-PCR. The serum content of carcinoembryonic antigen (CEA) was measured by Electrochemiluminescence immunoassay. The serum contents of PGI and PGII were measured by Enzyme linked immunosorbent assay. The diagnostic value of serum markers on AG and GC was analyzed by receiver operating characteristic (ROC) analysis.

**Results:**

The expression of miR-101-3p, the content of PGI and the ratio of PGI/II were significantly decreased, and the content of PGII was significantly increased in AG patients compared with those in normal controls. The changes of the above serum indicators were more obvious in GC patients than those in AG patients. The content of CEA was significantly higher in GC patients than that in AG patients. In addition, the expression of miR-101-3p was negatively associated with the submucosal infiltration in GC patients. MiR-101-3p exhibited high diagnostic value on AG (AUC 0.8493, sensitivity 80.33%, specificity 80%) and GC (AUC 0.8749, sensitivity 72.09%, specificity 86.49%). MiR-101-3p + PGI + PGI/II (AUC 0.856, sensitivity 80.23%, specificity 77.05%) exhibited a high diagnostic value in distinguishing between AG and GC.

**Conclusions:**

MiR-101-3p was a potential diagnostic marker for AG and GC. MiR-101-3p + PGI + PGI/II was effective in distinguishing between AG and GC.

## Background

Gastric cancer (GC), also known as stomach cancer, is the fifth most common malignant tumor and the third leading cause of cancer-related death worldwide [1]. In 2018, GC newly occurred in about 1,000,000 people and caused 783,000 deaths [1]. Early GC, whereby disease is limited to mucosa and submucosa, confers a 5-year survival rate of greater than 95% [2]. Most patients with early GC present with symptoms indistinguishable from benign peptic ulcer disease [3]. Screening for this group of patients improves detection rate of early GC and therefore its prognosis [4]. Although several screening approaches have been proposed, including indirect atrophy detection by measuring pepsinogen (PG) in the circulation, none of them have so far been implemented, and more study data is required to justify any implementation [5].

Cancer exhibits remarkable complexity at the molecular level that is associated with multiple genes, proteins, pathways and regulatory interconnections [6, 7]. MicroRNAs (miRNAs) are small, non-coding RNA molecules that are involved in the regulation of the tumorigenesis, progression, and prognosis of GC [8]. MiR-101 is known as a tumor suppressor in GC. Carvalho J et al. have confirmed that miR-101 is significantly down-regulated in GC tissues in comparison with normal gastric mucosas [9]. Dong X et al. have proved that the expression of miR-101 in GC tissues is significantly lower than that in cancer-adjacent normal tissues, and miR-101 is an independent prognostic factor affecting the overall survival of GC patients [10]. Rossi T et al. have shown that miR-101 is significantly down-regulated in 33 patients with intestinal GC [11]. In addition to that in tumor tissues, the expression of miR-101 in plasma is also a sensitive indicator for GC. Imamura T et al. have confirmed that low miR-101 plasma level is associated with advanced T factor, advanced disease stage, peritoneal metastasis, and poor prognosis in GC patients [12]. A small RNA sequencing spectra of plasma-derived exosomes has shown that miR-101-3p is a biomarker for GC with ovarian metastasis [13].

The serum parameters, PGI, PGII and PGI/II ratio are potential diagnostic indicators for atrophic gastritis (AG) and GC. Zoalfaghari A et al. have proved that the PGI and PGI/II ratio are significantly decreased in AG patients compared with the controls, and these two factors are potential biomarkers for screening AG with high sensitivity and specificity [14]. Ping L et al. have indicated that the serum PGI, PGII, and PGI/II ratio are closely related to the occurrence of GC and its precancerous disease [15]. Zhang XM et al. have confirmed that patients with early and advanced GC have a significantly lower PGI/II ratio than patients with AG [16]. Note worthily, miRNA-let-7 targeting pepsinogen C (PGC) is an important diagnostic indicator for AG and GC. Liu WJ et al. have demonstrated that the serum miRNA-let-7c is negatively correlated to the expression of PGC, and exhibits significant difference in the CON-AG-GC disease sequence [17]. Wu YF et al. have proved that miRNA-let-7e rs8111742 AA genotype increases the risk of GC in *H. pylori*-positive patients [18]. However, the diagnostic value of PG combined with miR-101 on AG and GC remains unclear.

In this study, the serum expression of miR-101-3p, and the serum contents of PGI, PGII and carcinoembryonic antigen (CEA) were measured in patients with AG and GC, and healthy volunteers. The diagnostic value of serum marker alone and combinations was analyzed. Our findings may reveal potential diagnostic markers for AG and GC.

## Methods

### Patients

A total of 61 AG patients and 86 GC patients (Chinese Han) were screened from the Department of Digestion, Dongying People’s Hospital between January 2018 and November 2018. AG and GC were confirmed by gastric biopsy. A total of 50 healthy volunteers were enrolled as the normal controls. There was no use of special medications (such as proton pump inhibitor and H2 receptor antagonist) at 1-week before admission. This study was approved by the Ethics Committee of our hospital (No: 2019136), and informed consents were obtained from all subjects.

### Serum collection

The venous blood (10 ml) was collected from participants in the morning, after overnight fasting. The blood was kept for 30 min at 25 °C (coagulation), and then centrifuged at 1500 rpm for 30 min, 3000 rpm for 5 min and 4500 rpm for 5 min at 25 °C. The supernatant (serum) was stored at − 80 °C until use.

### Quantitative real-time PCR (qRT-PCR)

The expression of miR-101-3p in the serum was detected by qRT-PCR. Total RNA was isolated from serum samples using a mirVana PARIS kit (Ambion, Austin, TX). The concentration and purity of isolated RNA were detected by an UV spectrophotometer (Bio-Rad, Hercules, CA, USA). RNA was subsequently reverse transcribed into cDNA using a RTSuperMix Kit (Vazyme, Nanjing, China) in accordance with the manufacturer’s instruction. The qRT-PCR was performed on a LightCycler (Roche Diagnostics, Basel, Switzerland) using specific primers (miR-101-3p-F: 5′-TGGGCTACAGTACTGTGATA-3′; miR-101-3p-R: 5′-TGCGTGTCGTGGAGTC-3′). U6 (U6-F: 5′-CATTGCACTTGTCTCGGTCT-3′; U6-R: 5′-GGTCCGAGGTATTCGCACT-3′) was used as an internal control. All primers were synthesized by Sangon Biotechnology Co., Ltd. (Shanghai, China). The qRT-PCR program included 94 °C for 10 min, 40 cycles of 94 °C for 45 s, 60 °C for 45 s, and 72 °C for 45 s. The relative expression level was calculated by 2^-∆∆CT^ method [19].

### Electrochemiluminescence immunoassay (ECLIA)

The serum content of CEA was measured using an ECL kit (Roche, Switzerland) in accordance with the manufacturer’s instruction. The ECLIA signal related to the CEA concentration was detected on a Roche Cobase E601 ECLIA instrument (Roche). The detection threshold of CEA was 6.5 ng/mL.

### Enzyme linked immunosorbent assay (ELISA)

The serum contents of PGI and PGII were measured using ELISA Kits (Huitai, Shanghai, China) in accordance with the manufacturers’ instructions. The optical density (OD) value at 450 nm was measured by a microplate reader. The contents of PGI and PGII were calculated according to the standard curves.

### Statistical analysis

Statistical analysis was performed using SPSS (version 18.0, Chicago, IL, USA). Quantitative data were expressed as mean ± standard deviation (SD), and the difference among different groups was analyzed by ANOVA, followed by Tukey’s multiple comparison test. Qualitative data were expressed as number, and the difference was analyzed by X2 test. The diagnostic value of variables was analyzed by receiver operating characteristics (ROC) analysis. Youden index was used to determine the optimal cutoff value (Cut off). A *P*-value of less than 0.05 was considered as statistically significant.

## Result

### The expression of miR-101-3p, and the contents of PGI, PGI/II and CEA in the serum of participants

The expression of miR-101-3p, and the contents of PGI, PGII and CEA were detected in the serum of participants. As shown in Table 1, the expression of miR-101-3p, the content of PGI and the ratio of PGI/II were significantly decreased in AG patients compared with those in normal controls (*P* <  0.05). The above parameters were significantly lower in GC patients than those in AG patients (*P* <  0.05). On the contrary, the content of PGII was significantly higher in AG patients than that in normal controls (*P* <  0.05). The contents of PGII and CEA were significantly higher in GC patients than those in AG patients (*P* <  0.05) (Table 1). The correlation between the expression of miR-101-3p and the baseline information of GC patients was further analyzed. As shown in Table 2, the expression of miR-101-3p was negatively associated with the submucosal infiltration in GC patients (*P* = 0.0299). There was no significantly correlation between the expression of miR-101-3p and the age, sex, differentiation, *H. pylori* infection and histological type.

**Table 1:** The expression of miR-101-3p, and the contents of PGI, PGII and CEA in the serum of patients with atrophic gastritis (AG) and gastric cancer (GC), and healthy volunteers (Normal control)

Index	Normal control (*n* = 50)	AG (*n* = 61)	GC (*n* = 86)
Sex			
Male, N (%)	26 (52)	33 (54.1)	36 (41.9)
Female, N (%)	24 (48)	28 (45.9)	50 (58.1)
Age (years)	45.150 ± 1.400	46.220 ± 1.600	60.110 ± 2.100
miR-101-3p	1.000 ± 0.145	0.816 ± 0.068*	0.730 ± 0.064*#
PGI (μg/l)	75.460 ± 12.480	66.770 ± 9.240*	58.490 ± 11.950*#
PGII (μg/l)	11.000 ± 3.380	14.870 ± 9.090*	17.790 ± 16.010*#
PGI/II ratio	7.350 ± 1.970	5.440 ± 2.040*	4.220 ± 1.710*#
CEA (μg/l)	1.850 ± 0.730	2.310 ± 1.080	3.660 ± 1.380*#

Notes: *PG* Pepsinogen, *AG* Atrophic gastritis, *GC* Gastric cancer, *CEA* Carcinoembryonic antigen; Quantitative data were expressed as mean ± standard deviation (SD), and the difference was analyzed by ANOVA, followed by Tukey’s multiple comparison test. Qualitative data were expressed as number (percentage), and the difference was determined by X2 test. *, *P* <  0.05 compared with normal control; #, *P* <  0.05 compared with AG

**Table 2:** The correlation between the expression of miR-101-3p and the baseline information of patients with gastric cancer (GC)

Index	Total cases	miR-101-3p expression		*P*-value
		High	low	
Age				0.3722
< 60 years	32	18	14	
≥ 60 years	54	25	29	
Sex				0.8123
Male	61	30	31	
Female	25	13	12	
Differentiation				0.2788
Middle/high	39	22	17	
Low	47	21	26	
Infiltration				0.0299*
Intramucosal	48	29	19	
Submucosal	38	14	24	
*H. pylori* infection				0.3864
*H. Pylori* (−)	47	21	26	
*H. Pylori* (+)	39	22	17	
Histological type				0.1600
Adenocarcinoma	60	26	34	
Mucinous carcinoma	3	1	2	
Signet ring cell carcinoma	8	5	3	

Notes: High or low expression was defined according to the median expression of miR-101-3p. Qualitative data were expressed as number, and the difference was determined by X2 test. *, *P* <  0.05

### The diagnostic value of serum marker alone on AG and GC

**Fig. 1:**
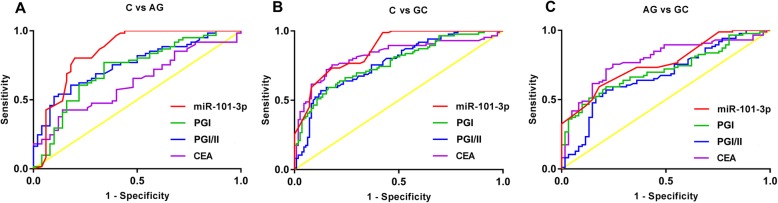
The diagnostic value of serum miR-101-3p, PGI, and PGI/II alone on AG and GC was analyzed by ROC analysis. **a**, the ROC curves of serum markers between normal controls (C) and AG patients; **b**, the ROC curves of serum markers between normal controls (C) and GC patients; **c**, the ROC curves of serum markers between AG and GC patients

The ROC curves were established to evaluate the diagnostic value of miR-101-3p, PGI, PGI/PGII, and CEA alone on AG and GC. As shown in Table 3, miR-101-3p (AUC 0.8493, sensitivity 80.33%, specificity 80%) exhibited a high diagnostic value on AG. The diagnostic value of CEA on AG was relatively low, and the sensitivity was only 41.62% (Fig. 1a). A high diagnostic value on GC was observed in miR-101-3p (AUC 0.8749, sensitivity 72.09%, specificity 86.49%) and CEA (AUC 0.8235, sensitivity 75.58%, specificity 81.98%). The diagnostic value of PGI and PGI/II on GC was limited due to the low sensitivity (PGI 59.3%, PGI/II 56.98%) (Fig. 1b). In addition, miR-101-3p and CEA were limited in distinguishing between AG and GC, and the sensitivity and specificity were all less than 80% (Fig. 1c). The detail information of each marker was listed in Table 3.

**Table 3:** The diagnostic value of serum marker alone on atrophic gastritis (AG) and gastric cancer (GC)

	Cut off	Sensitivity	Specificity	AUC	95% CI	*P*-value
C vs. AG	miR-101-3*p* < 0.8765	80.33%	80.00%	0.8493	0.771–0.928	< 0.0001
	PG I < 71.56 (μg/l)	77.05%	66.00%	0.7187	0.621–0.816	< 0.0001
	PG I/II ratio < 5.6	60.66%	82.00%	0.7549	0.666–0.844	< 0.0001
	CEA > 2.52 (ng/ml)	41.62%	86.00%	0.6289	0.526–0.732	0.0198
C vs. GC	miR-101-3*p* < 0.759	72.09%	86.49%	0.8749	0.829–0.921	< 0.0001
	PG I < 59.98 (μg/l)	59.3%	83.78%	0.7702	0.704–0.836	< 0.0001
	PG I/II ratio < 4.22	56.98%	87.39%	0.7697	0.704–0.835	< 0.0001
	CEA > 2.875 (ng/ml)	75.58%	81.98%	0.8235	0.760–0.887	< 0.0001
AG vs. GC	miR-101-3*p* < 0.756	68.60%	77.05%	0.7710	0.697–0.845	< 0.0001
	PG I < 58.22 (μg/l)	51.16%	86.89%	0.7130	0.631–0.795	< 0.0001
	PG I/II ratio < 4.01	52.33%	83.61%	0.6760	0.587–0.764	< 0.0001
	CEA > 2.875 (ng/ml)	75.58%	75.41%	0.7850	0.710–0.860	< 0.0001

Notes: *AUC* Area-under-the-curve, *CI* Confidence intervals, *PG* Pepsinogen, *AG* Atrophic gastritis, *GC* Gastric cancer, *C* Normal control. The diagnostic value of variables was analyzed by receiver operating characteristics (ROC) analysis. Youden index was used to determine the optimal cutoff value (Cut off). A *P* <  0.05 was considered as statistically significant

### The diagnostic value of serum markers combination on AG and GC

**Fig. 2:**
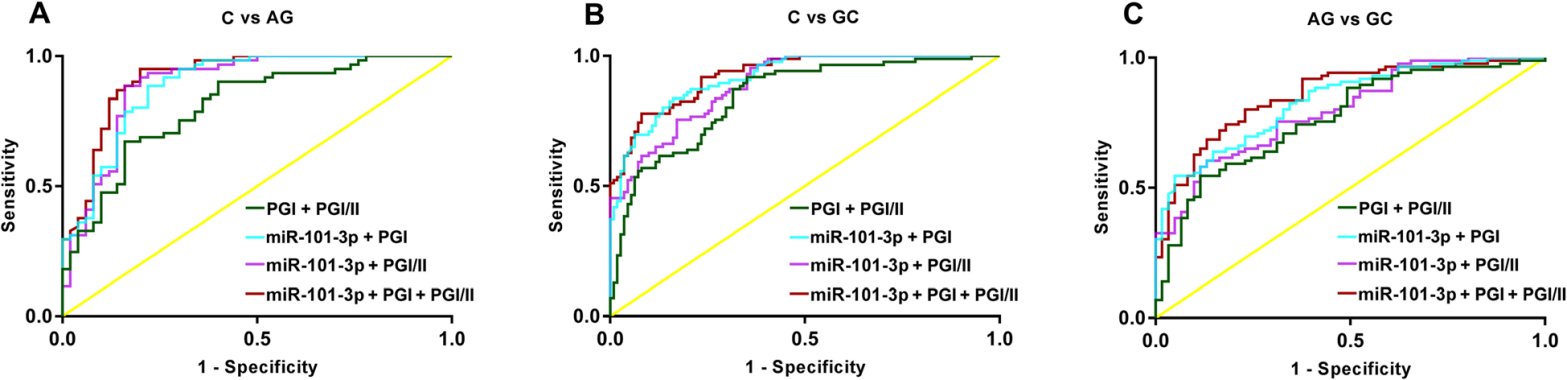
The diagnostic value of serum marker combinations on AG and GC was analyzed by ROC analysis. **a**, the ROC curves of the combinations between normal controls (C) and AG patients; **b**, the ROC curves of the combinations between normal controls (C) and GC patients; **c**, the ROC curves of the combinations between AG and GC patients

MiR-101-3p, PGI and PGI/II were combined to diagnose AG and GC. As shown in Table 4, miR-101-3p + PGI/II (AUC 0.892, sensitivity 88.52%, specificity 84.00%) and miR-101-3p + PGI + PGI/II (AUC 0.917, sensitivity 95.08%, specificity 80.00%) exhibited high diagnostic value on AG. The diagnostic value of PGI + PGI/II on AG was limited due to low sensitivity (67.21%), and the diagnostic value of miR-101-3p + PGI on AG was limited due to low specificity (64.04%) (Fig. 2a). In diagnosis of GC, PGI + PGI/II was limited due to low specificity (64.86%). Three miR-101-3p combinations all exhibited high diagnostic value on GC (AUC > 0.891). However, the sensitivity of miR-101-3p + PGI + PGI/II (77.91%) was not satisfied (Fig. 2b). In addition, only miR-101-3p + PGI + PGI/II (AUC 0.856, sensitivity 80.23%, specificity 77.05%) exhibited high diagnostic value in distinguishing between AG and GC. The diagnostic value of the other three combinations was limited due to low sensitivity (< 61%) (Fig. 2c). The detail information of each combination was listed in Table 4.

**Table 4:** The diagnostic value of serum marker combinations on atrophic gastritis (AG) and gastric cancer (GC)

		Sensitivity	Specificity	AUC	95% CI	*P* value
C vs. AG	PG I + PG I/II	67.21%	84.00%	0.807	0.727–0.888	< 0.0001
	miR-101-3p + PG I	88.52%	64.04%	0.890	0.828–0.952	< 0.0001
	miR-101-3p + PG I/II	88.52%	84.00%	0.892	0.827–0.957	< 0.0001
	miR-101-3p + PG I + PG I/II	95.08%	80.00%	0.917	0.863–0.972	< 0.0001
C vs. GC	PG I + PG I/II	91.86%	64.86%	0.842	0.787–0.897	< 0.0001
	miR-101-3p + PG I	83.72%	83.78%	0.918	0.882–0.954	< 0.0001
	miR-101-3p + PG I/II	95.35%	88.52%	0.891	0.849–0.933	< 0.0001
	miR-101-3p + PG I + PG I/II	77.91%	91.89%	0.928	0.895–0.961	< 0.0001
AG vs. GC	PG I + PG I/II	54.65%	88.52%	0.768	0.691–0.844	< 0.0001
	miR-101-3p + PG I	54.65%	95.08%	0.833	0.770–0.897	< 0.0001
	miR-101-3p + PG I/II	60.47%	86.89%	0.798	0.728–0.868	< 0.0001
	miR-101-3p + PGI + PG I/II	80.23%	77.05%	0.856	0.795–0.916	< 0.0001

Notes: *AUC* Area-under-the-curve, *CI* confidence intervals, *PG* Pepsinogen, *AG* Atrophic gastritis, *GC* Gastric cancer, *C* Normal control. The diagnostic value of variables was analyzed by receiver operating characteristics (ROC) analysis. Youden index was used to determine the optimal cutoff value (Cut off). A *P* < 0.05 was considered as statistically significant

## Discussion

GC is a common cancer that associated with high morbidity and mortality. Because early GC exhibits no specific symptoms, GC is usually diagnosed at a late stage, leading to poor prognosis [20]. Early diagnosis of GC may greatly improve the survival rate of GC patients [21]. In this study, the diagnostic value of serum miR-101-3p and its combinations on AG and GC was analyzed. Our results showed that the serum expression of miR-101-3p was significantly lower in AG patients than that in normal controls, and was significantly lower in GC patients than that in AG patients. MiR-101-3p exhibited high diagnostic value on AG and GC. The combination of miR-101-3p, PGI and PGI/II exhibited a high diagnostic value in distinguishing between AG and GC.

The dysregulation of miRNAs can promote the cell-cycle progression, confer the resistance to apoptosis, and enhance the invasiveness and metastasis of tumor cells [22]. Previous studies have proved that some miRNAs are potential prognostic factors of GC. Nishida N et al. have indicated that miR-125a-5p is an independent prognostic factor for the survival of GC patients [23]. Inoue T et al. have proved that miR-107 is an independent prognostic factor for the overall survival rate and disease-free survival rate of GC patients [24]. Naito Y et al. have demonstrated that miR-145 is a potential prognostic factor of scirrhous-type GC [25]. MiR-101 acts as a tumor suppressor in GC. The expression of miR-101 in GC tissues was significantly decreased compared with adjacent normal tissues [9–11]. Dong X et al. have proved that miR-101 is an independent prognostic factor for the overall survival of GC patients that correlated with the pathological differentiation degree, lymph node metastasis and depth of infiltration [10]. Imamura T et al. have confirmed that plasma miR-101 is a biomarker for GC that associated with the T factor, disease stage, peritoneal metastasis and the prognosis [12]. In this study, we found that the serum miR-101-3p was significantly decreased in GC patients compared with normal controls. The expression of miR-101-3p was negatively associated with the submucosal infiltration in GC patients. Our findings are consistent with previous studies, and illustrate that miR-101-3p is a potential diagnostic factor for GC. ROC curves were then established to evaluate the diagnostic value of serum miR-101-3p on AG and GC. The results showed that miR-101-3p exhibited a high diagnostic value on AG (AUC 0.8493, sensitivity 80.33%, specificity 80%) and GC (AUC 0.8749, sensitivity 72.09%, specificity 86.49%). However, miR-101-3p was limited in distinguishing between AG and GC due to relatively low sensitivity and specificity (all < 80%). All these findings indicate that the serum miR-101-3p can be used in the diagnosis of AG and GC with high sensitivity and specificity.

Serum PG can reflect the histological condition of gastric mucosa, which exhibits potential diagnostic value for AG and GC [26]. Serum PG test is introduced for mass screening to identify individuals at high risk for GC [27]. Begum A et al. have proved that the serum PGI/II ratio is effective in the diagnosis of GC with high sensitivity (70.0%), specificity (97.5%), and accuracy (83.8%) [28]. Actually, the combination of serum PG and other indicators is successfully used for the prediction of GC [29]. Sembiring J et al. have shown that PGI combined with CEA can be used for the diagnosis of GC from non-GC individuals with a sensitivity of 94.1% and a specificity of 80% [30]. Niu WW et al. have indicated that the sensitivity and specificity of miR-92a combined with PG on GC is 86.49 and 89.32%, respectively [31]. Wu G et al. have proved that the combination of gastrin-17 and PGI/II ratio exhibits high sensitivity (96.2%) and accuracy (86.2%) in screening of GC [32]. In this study, miR-101-3p + PGI/II and miR-101-3p + PGI + PGI/II exhibited a high diagnostic value on AG, and miR-101-3p + PGI and miR-101-3p + PGI/PGII exhibited a high diagnostic value on GC. These results indicate that the addition of PG further improves the diagnostic value of miR-101-3p on AG and GC. Note worthily, miR-101-3p + PGI + PGI/II (AUC 0.856, sensitivity 80.23%, specificity 77.05%) exhibited a high diagnostic value in distinguishing between AG and GC. This phenomenon indicates that the combination of PG greatly improves the low sensitivity and specificity of miR-101-3p in distinguishing between AG and GC.

Our study exhibits some limitations. First, the insufficient subjects may not reflect the accurate diagnostic value of miR-101-3p and its combinations in clinical practice. Second, a targeted approach rather than hypothesis free approaches such as microarray assays or sequencing platform limits the discovery of novel miRNA markers. Further researches on these fields are still needed.

## Conclusions

In conclusion, the serum levels of miR-101-3p, PGI, PGII, and PGI/II were significantly changed in AG and GC. The expression of miR-101-3p was negatively associated with the submucosal infiltration in GC patients. MiR-101-3p exhibited a high diagnostic value on AG and GC. MiR-101-3p + PGI + PGI/II exhibited a high diagnostic value in distinguishing between AG and GC. MiR-101-3p and its combinations may be used as potential markers for early diagnosis of GC.

## Data Availability

The datasets used and/or analysed during the current study are available from the corresponding author on reasonable request.

## Abbreviations

AG: Atrophic gastritis
CEA: Carcinoembryonic antigen
ECLIA: Electrochemiluminescence immunoassay
ELISA: Enzyme linked immunosorbent assay
GC: Gastric cancer
PG: Pepsinogen
ROC: Receiver operating characteristic

## References

[1] Bray F, Ferlay J, Soerjomataram I, Siegel RL, Torre LA, Jemal A (2018). Global cancer statistics 2018: GLOBOCAN estimates of incidence and mortality worldwide for 36 cancers in 185 countries. CA Cancer J Clin.

[2] Song Z, Wu Y, Yang J, Yang D, Fang X (2017). Progress in the treatment of advanced gastric cancer. Tumour Biol.

[3] Sugimoto M, Yamaoka Y, Furuta T (2010). Influence of interleukin polymorphisms on development of gastric cancer and peptic ulcer. World J Gastroenterol.

[4] Cong AW, Sun YH, Shen ZB, Wang XF, Yin YQ, Fang Y, et al. Relationship between clinicopathologic factors and prognosis of patients with early gastric cancer. Chin J Digest Surg. 2009;8:338–40.

[5] Pasechnikov V, Chukov S, Fedorov E, Kikuste I, Leja M (2014). Gastric cancer: Prevention,screening and early diagnosis. World J Gastroenterol.

[6] Lizbeth RK, Augustine D, Rao RS, Sowmya SV, Patil S (2017). Biomarkers in tumorigenesis using Cancer cell lines: a systematic review. Asian Pac J Cancer Prev.

[7] Nagaraj SH, Reverter A (2011). A Boolean-based systems biology approach to predict novel genes associated with cancer: application to colorectal cancer. BMC Syst Biol.

[8] Tetsuya U, Stefano V, Hiroshi O, Masayoshi S, Cristian T, Simona R (2010). Relation between microRNA expression and progression and prognosis of gastric cancer: a microRNA expression analysis. Lancet Oncol.

[9] Carvalho J, van Grieken NC, Pereira PM, Sousa S, Tijssen M, Buffart TE (2012). Lack of microRNA-101 causes E-cadherin functional deregulation through EZH2 up-regulation in intestinal gastric cancer. J Pathol.

[10] Dong X, Liu Y (2018). Expression and significance of miR-24 and miR-101 in patients with advanced gastric cancer. Oncol Lett.

[11] Rossi Tania, Tedaldi Gianluca, Petracci Elisabetta, Abou Khouzam Raefa, Ranzani Guglielmina Nadia, Morgagni Paolo, Saragoni Luca, Monti Manlio, Calistri Daniele, Ulivi Paola, Molinari Chiara (2019). E-cadherin Downregulation and microRNAs in Sporadic Intestinal-Type Gastric Cancer. International Journal of Molecular Sciences.

[12] Imamura T, Komatsu S, Ichikawa D, Miyamae M, Okajima W, Ohashi T (2017). Low plasma levels of miR-101 are associated with tumor progression in gastric cancer. Oncotarget..

[13] Zhang Y, Han T, Feng D, Li J, Wu M (2019). Peng X, et al.

[14] Zoalfaghari A, Aletaha N, Roushan N, Taslimi R, Foroutan H, Faridnia B (2014). Accuracy of pepsinogens for early diagnosis of atrophic gastritis and gastric cancer in Iranian population. Med J Islam Repub Iran.

[15] Li P, He C, Sun L, Dong N, Yuan Y. Pepsinogen I and II expressions in situ and their correlations with serum pesignogen levels in gastric cancer and its precancerous disease. BMC Clin Pathol. 2013;13:22. 10.1186/1472-6890-13-22 PMC3846408 24004680

[16] Zhang XM, Li JX, Zhang GY, Li XH, Gu H (2014). The value of serum pepsinogen levels for the diagnosis of gastric diseases in Chinese Han people in midsouth China. BMC Gastroenterol.

[17] Liu WJ, Xu Q, Sun LP, Dong QG, He CY, Yuan Y (2015). Expression of serum let-7c, let-7i, and let-7f microRNA with its target gene, pepsinogen C, in gastric cancer and precancerous disease. Tumour Biol.

[18] Wu YF, Xu Q, He CY, Li Y, Liu JW, Deng N (2017). Association of Polymorphisms in three pri-miRNAs that Target Pepsinogen C with the Risk and Prognosis of Gastric Cancer. Sci Rep.

[19] Livak KJST (2001). Analysis of Relative Gene Expression Data Using Real-Time Quantitative PCR and the 2(−Delta Delta C(T))Method. Methods.

[20] Walker R, Poleszczuk J, Mejia J, Lee JK, Coppola D (2017). Toward early detection of helicobacter pylori-associated gastric cancer. Gastric Cancer.

[21] Tan Yih K., Fielding John W.L. (2006). Early diagnosis of early gastric cancer. European Journal of Gastroenterology & Hepatology.

[22] Wu WKK, Lee CW, Cho CH, Fan D, Wu K, Yu J (2010). MicroRNA dysregulation in gastric cancer: a new player enters the game. Oncogene..

[23] Nishida N, Mimori K, Fabbri M, Yokobori T, Sudo T, Tanaka F (2011). MicroRNA-125a-5p is an independent prognostic factor in gastric cancer and inhibits the proliferation of human gastric cancer cells in combination with trastuzumab. Clin Cancer Res.

[24] Inoue T, Iinuma H, Ogawa E, Inaba T, Fukushima R (2012). Clinicopathological and prognostic significance of microRNA-107 and its relationship to DICER1 mRNA expression in gastric cancer. Oncol Rep.

[25] Naito Y, Yasuno K, Tagawa H, Sakamoto N, Oue N, Yashiro M (2014). MicroRNA-145 is a potential prognostic factor of scirrhous type gastric cancer. Oncol Rep.

[26] Zhao J, Zhu C, Li Z. Diagnostic value of serum pepsinogen, gastrin-17 and helicobacter pylori antibody for atrophic gastritis and gastric cancer. Chin J Gastroenterol. 2016;21:376–9.

[27] Miki K (2006). Gastric cancer screening using the serum pepsinogen test method. Gastric Cancer.

[28] Begum A, Baten MA, Begum Z, Ahsan MM, Rahman SF, Chowdhury F (2017). Role of serum pepsinogen I and II ratio in screening of gastric carcinoma. Mymensingh Med J.

[29] Watabe H, Mitsushima T, Yamaji Y, Okamoto M, Wada R, Kokubo T (2005). Predicting the development of gastric cancer from combining Helicobacter pylori antibodies and serum pepsinogen status: a prospective endoscopic cohort study. Gut.

[30] Sembiring J, Sarumpaet K, Ganie RA (2018). Diagnostic test pepsinogen I and combination with tumor marker CEA in gastric cancer.

[31] Niu WW, Yang CC, Duan ZY, Huo XH, Gastroenterology DO. The diagnostic value of miRNA-92a combined with micro pepsinogen in gastric carcinoma. J Hebei Med Univ. 2017;38:638–41.

[32] Wu G, Chen L, Zhao C, Xiao M (2017). The value of combined detection of serum gastrin-17 and pepsinogen in the early screening of gastric cancer. Biomed Res.

